# Determining the Drivers of Academic Success in Surgery: An Analysis of 3,850 Faculty

**DOI:** 10.1371/journal.pone.0131678

**Published:** 2015-07-15

**Authors:** Nakul P. Valsangkar, Teresa A. Zimmers, Bradford J. Kim, Casi Blanton, Mugdha M. Joshi, Teresa M. Bell, Attila Nakeeb, Gary L. Dunnington, Leonidas G. Koniaris

**Affiliations:** Department of Surgery, Indiana University School of MedicineIndianapolis, IN, 46202, United States of America; University of Florida, UNITED STATES

## Abstract

**Objective:**

Determine drivers of academic productivity within U.S. departments of surgery.

**Methods:**

Eighty academic metrics for 3,850 faculty at the top 50 NIH-funded university- and 5 outstanding hospital-based surgical departments were collected using websites, Scopus, and NIH RePORTER.

**Results:**

Mean faculty size was 76. Overall, there were 35.3% assistant, 27.8% associate, and 36.9% full professors. Women comprised 21.8%; 4.9% were MD-PhDs and 6.1% PhDs. By faculty-rank, median publications/citations were: assistant, 14/175, associate, 39/649 and full-professor, 97/2250. General surgery divisions contributed the most publications and citations. Highest performing sub-specialties per faculty member were: research (58/1683), transplantation (51/1067), oncology (41/777), and cardiothoracic surgery (48/860). Overall, 23.5% of faculty were principal investigators for a current or former NIH grant, 9.5% for a current or former R01/U01/P01. The 10 most cited faculty (MCF) within each department contributed to 42% of all publications and 55% of all citations. MCF were most commonly general (25%), oncology (19%), or transplant surgeons (15%). Fifty-one-percent of MCF had current/former NIH funding, compared with 20% of the rest (p<0.05); funding rates for R01/U01/P01 grants was 25.1% vs. 6.8% (p<0.05). Rate of current-NIH MCF funding correlated with higher total departmental NIH rank (p < 0.05).

**Conclusions:**

Departmental academic productivity as defined by citations and NIH funding is highly driven by sections or divisions of research, general and transplantation surgery. MCF, regardless of subspecialty, contribute disproportionally to major grants and publications. Approaches that attract, develop, and retain funded MCF may be associated with dramatic increases in total departmental citations and NIH-funding.

## Introduction

Success for individual faculty in academic surgery, like in other medical specialties, may be measured by numbers of publications, citations, and external research funding, especially from the National Institutes of Health (NIH) [1,2]. These measures are validated, impartial metrics of academic productivity that are considered amongst the best measures of individual academic accomplishment [3,4][5][6,7]. Furthermore, such academic metrics are frequently considered in a number of other situations including the determination of academic promotion and entry into academic organizations [8–11][12]. Similarly, derived journal metrics which use the same data are highly respected measures for journal impact and significance [10,12–15]. Authors rely heavily on such journal metrics when choosing where to report findings. Most journals also emphasize their relative metrics when compared to other journals [16][17].

Aggregate and ranked metrics are available for journals by specialty. However, to date, little work has been done to determine metrics of faculty academic productivity within specific disciplines. At present, limited examinations focused mostly on publications and citations have been reported in subsets of physicians in relatively few specialties [18–20][21,22][23,24][20,25][20,26]. To date, no such examination has been done within the field of surgery.

We sought to delineate aggregate academic metrics for surgical faculty by specialty and department in the field of surgery, sub-specialties within surgery and identify the individuals who function as drivers of academic success. We also hoped to determine potential methods to quantify academic strengths and weaknesses in specific surgical sections, divisions or departments. Such an understanding would be useful in implementing strategies to improve overall academic performance, and retain high performing faculty.

We anticipate that such data could also be used as a benchmark to compare individual academic accomplishments with aggregate faculty peers both within and across specialties. Such metrics could inform situations such as consideration for promotion, potential new positions, selection for additional funding, and to help better identify the subset of faculty with the greatest promise for future academic success.

In order to determine quantitative measures of academic accomplishments we: (1) Identified the top departments of surgery in the U.S. based upon total, current NIH grants. (2) Determined demographic and individual academic metrics including faculty rank, specialty, publications, citations, H-index and extramural NIH funding. (3) Generated aggregate data by a number of academic metrics to help identify potential drivers of academic success within specialties and by departments. (4) Determined which faculty within a department drive academic productivity. Herein, we report summary statistics for this examination and introduce the observation that a small group of faculty greatly drive overall academic productivity for a department.

## Materials and Methods

In order to define the academic drivers of success in the top U.S. departments of surgery, the top 50-ranked-university based departments of surgery were identified based on current NIH funding available from the Blue Ridge Institute for Medical Research [27]. Additionally, a Medline search and review of current meetings was performed to identify additional institutions that had a significant academic impact but not present on the NIH funding rank list. This search yielded 5 additional hospital-based departments of surgery all of which are associated with, but separate, from a medical school. These 55 departments of surgery were then organized by rank based on the NIH funding received by the department of surgery, and then were compiled into a master database of 55 departments of surgery. Online websites for each of the 55 identified departments of surgery were then used to generate a list of surgical faculty members at these institutions. Using this algorithm, 3,850 surgical faculty were identified including surgeons and research faculty. Demographic variables including: academic degrees, academic rank, the career track–clinical or research, specialty, division, and whether or not the faculty held a title such as division chief, or chairman/chairwoman, were collected from the departmental websites as available.

**Fig 1:**
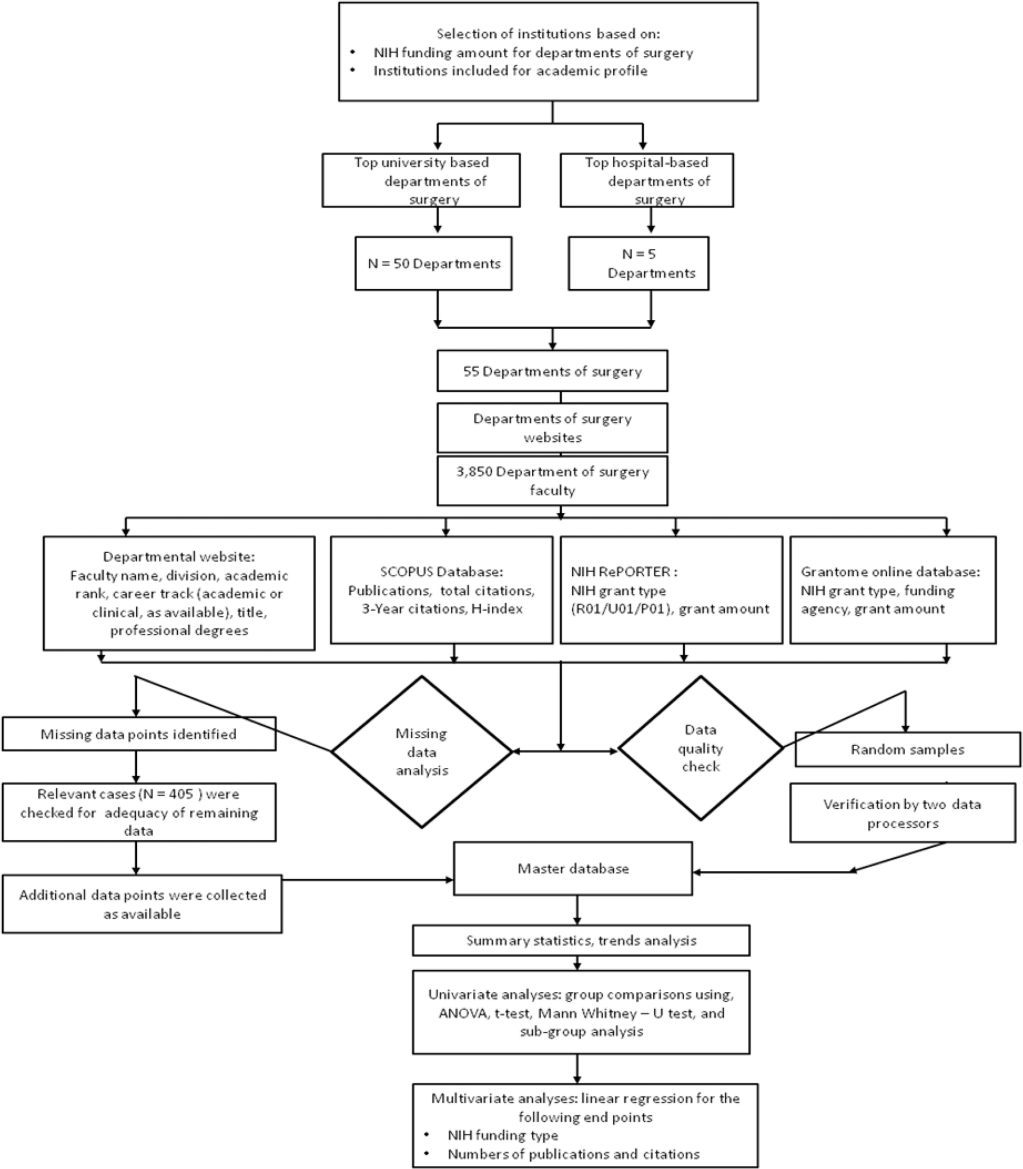
Study design flowchart.

Three additional data sources, as indicated in **Fig 1,** were used to collect additional data for the 3,850 surgical faculty: 1) Elsevier’s SCOPUS bibliographical database (http://proxyauth.uits.iu.edu/auth/ulib.pl?url=http://www.scopus.com) 2) the NIH Research Portfolio Online Reporting Tools (RePORT) (http://report.nih.gov/) and 3) Grantome (http://grantome.com/) databases for the type and number of NIH grants awarded to each of these faculty.

### Scopus

For each faculty member identified the SCOPUS database was to determine their individual scholarly metrics including the total publications, total career citations, 3-year citations and H-index. SCOPUS was accessed online at http://scopus.com.proxy.medlib.iupui.edu. For all 3,850 faculty data that were collected, data collection occurred from 9/01/2014 through 1/31/2015.

### NIH funding

For all faculty identified in the database, data regarding research funding from the National Institutes of Health (NIH) was also collected. This data was searched from the NIH online data repository of funding, NIH RePORT and checked with the Grantome online database. These databases were used to collect data regarding the type of NIH funding, current (2014) funding dollar amounts, the total funding amount in dollars, the type of NIH grant (R01, U01, F32 etc), the funding agency (NCI, NAI, NIGMS etc), and the numbers of each of the NIH grants. These data were then used to create a binned variable to categorize NIH funding. The bins that were created included the following categories: (1) no current/former NIH funding, (2) NIH R01/U01/P01 funding, and (3) NIH smaller grants (F32, R03, T32, R23…) funding.

### Ethics statement

Only publically available data sets were queried for examination. This study was exempt from review by the Institutional Review Board (IRB) of Indiana University School of Medicine. (http://www.hhs.gov/ohrp/policy/checklists/decisioncharts.html).

### Database and Statistical Analysis

Data from each of the sources was collated into the master database. This database is available as the supporting information (S1 File). The variables in the database were categorized as either continuous or categorical. Continuous variables included, total numbers of publications, total career citations, 3-year citations, H-indices, and rank of the institution by total NIH funding amount for the department of surgery. Institutions were then grouped into quintiles based on department of surgery NIH funding. The rank bins were numbers 1–10, 11–20, 21–30, 31–40, and 41–50. The 5 hospital based divisions were excluded from the rank bins. Categorical variables included academic rank, divisions, credentials, gender, type of NIH funding, presence of current NIH funding, and rank group of the institution by NIH funding.

To summarize the data, trends analysis, by deciles of NIH funding rank and descriptive statistics were performed. Median and standard deviations were calculated for total publications, total- and three-year citations, and H-indices. For these variables, group comparisons were performed across the different categorical variables. Continuous variables were compared with t-test of means for two groups, and ANOVA for multiple group comparisons. Differences between categorical variables were tested using χ^2^ test and Mann-Whitney U test, as appropriate. Statistical tests with p < 0.05 was deemed significant. All statistical tests were performed using SPSS for Windows, Version 15.0. Chicago, IL, SPSS Inc. All statistical analyses were performed with consultation and input from a biostatistician (TB).

## Results

### Overview of academic productivity at the top NIH funded departments of surgery

Analysis of the dataset of 3,850 surgical faculty members at the 55 departments of surgery revealed median publications ± standard deviation (SD) of 35 ± 89. Median ± SD total citations for faculty were 581 ± 3005, of which 173 ± 792 were 3-year citations **(Table 1)** and these corresponded to a median (± SD) h-index of 11 ± 11. An approximately equal distribution of academic rank was observed with 35.3% being assistant, 27.8% being associate and 36.9% being full professors. There was a step-wise increase in both the numbers of publications and corresponding citations with progression in academic rank. The median publications ± SD and citations ± SD were: 1) assistant professors were 14 ± 31, 175 ± 617, 2) associate professors 39 ± 43, 649 ± 1778, and 3) full professors 97 ± 125, 2250 ± 4370.

**Table 1:** General and demographic characteristics of academic faculty from 55 departments of surgery. Cardiothoracic surgery includes cardiac and thoracic surgery. General surgery includes acute care surgery, general and minimally invasive surgery, surgical oncology, and trauma and critical care.

Parameter		n	(%)	Publications, Median ± SD (Range)	Citations, Median ± SD (Range)	3-year citations,Median ± SD(Range)	H index,Median ± SD
Surgical faculty, n		3,850	100%	35 ± 89 (1–1938)	581 ± 3005 (1–55118)	173 ± 792 (1–19986)	11 ± 11
Academic Ranks	Assistant Professor	1,359	35.3%	14 ± 31 (1–439)	175 ± 617 (1–6374)	64 ± 218 (1–2448)	6 ± 6
	Associate Professor	1071	27.8%	39 ± 43 (1–456)	649 ± 1778 (1–26158)	197 ± 499 (1–6361)	12 ± 8
	Professor	1,420	36.9%	97 ± 125 (1–1938)	2250 ± 4370 (2–55118)	491 ± 1196 (2–19986)	22 ± 13
Divisions	**Cardiothoracic Surgery**	400	10.4%	48 ± 88 (2–485)	860 ± 3198 (1–26158)	221 ± 793 (1–6361)	13 ± 12
	Cardiac	146	3.8%	54 ± 87 (2–439)	860 ± 3808 (3–26158)	225 ± 939 (3–6361)	14 ± 13
	Thoracic	254	6.6%	42 ± 89 (2–485)	855 ± 2869 (1–19664)	211 ± 714 (1–5463)	13 ± 11
	**General Surgery**	1,875	48.7%	34 ± 79 (1–636)	553 ± 3170 (1–36390)	188 ± 833 (1–7797)	11 ± 12
	Acute Care Surgery	89	2.3%	24 ± 53(1–245)	451 ± 1460 (11–5628)	188 ± 440 (5–1723)	11 ± 10
	General, minimally Invasive Surgery	1028	26.7%	33 ± 76 (2–636)	589 ± 2922 (1–36390)	160 ± 727 (1–7640)	11 ± 11
	Surgical Oncology	454	11.8%	41 ± 90 (1–488)	777 ± 3969 (1–27709)	265 ± 1080 (1–7797)	13 ± 15
	Trauma/Critical Care	304	7.9%	20 ± 95 (1–1002)	334 ± 2866 (2–26119)	12 ± 652 (1–5198)	8 ±10
	Pediatric Surgery	370	9.6%	34 ± 50 (2–264)	559 ± 1202 (1–8104)	138 ± 302 (1–2105)	10 ± 8
	Plastic Surgery	374	9.7%	24 ± 90 (1–1002)	327 ± 2158 (2–26119)	98 ± 494 (1–5198)	9 ± 8
	Science/Research	150	3.9%	58 ± 64 (3–337)	1683 ± 2315 (15–14101)	329 ± 628 (11–3198)	22 ± 10
	Transplant	392	10.2%	51 ± 162 (1–1938)	1067 ± 4696 (4–55118)	334 ± 685 (2–5452)	15 ± 13
	Vascular surgery	289	7.5%	33 ± 68 (2–439)	533 ± 2116 (2–14286)	147± 409 (1–2413)	10 ± 11
Academic credentials	M.D.	3,262	89%	33 ± 89 (1–1938)	517 ± 3027 (1–55118)	152 ± 791 (1–19986)	10 ± 11
	Ph.D.	224	6.1%	50 ± 86 (1–636)	1298 ± 2817 (1–26315)	353 ± 777(1–6293)	16 ± 12
	M.D., Ph.D.	178	4.9%	63 ± 123 (1–954)	1345 ± 4067 (2–33282)	424 ± 906 (15–4823)	17 ± 14

Analysis of the academic productivity by surgical divisions also indicated significant variation between specialties. Specialists in research divisions, transplant surgery cardiothoracic surgery, and surgical oncology were the most successful concerning their research productivity. The median publications ± SD and citations ± SD for these specialties were; science/research divisions: 58 ± 64, 1683 ± 2315, transplant surgery: 51 ± 162, 1067 ± 4696, cardiothoracic surgery: 48 ± 88, 860 ± 3198, and surgical oncology; 41 ± 90, 777 ± 3969 **(Table 1).**

### Impact of PhD degrees on surgical faculty academic metrics (Table 2)

Of the surgical faculty 6.1% had a Ph.D. without M.D., and 4.9% had an MD., Ph.D. **(Table 1).** Furthermore, 9.9% of the assistant, 12.4% of the associate and 14.7% of the full professors were identified as having a Ph.D. degree (Ph.D. or M.D., Ph.D.) In addition to research faculty, cardiothoracic surgeons and transplant surgeons were most likely to have a PhD. Depending on the academic rank, percentage (%) faculty with PhD or MD-PhD degrees were; cardiothoracic surgery 13.4%–16.5%, research faculty 75%–89%, and transplant surgery 12.1%–20.5%. There was considerable variation in fraction of Ph.D. faculty between academic specialty and depending on the academic rank **(Table 2).**

**Table 2:** Subset analysis of scholarly output by academic rank and credentials.

Parameter	N	(%)	Assistant Professor		Associate Professor		Professor	
			N = 1,359		N = 1071		N = 1,420	
(within entire dataset)			MD	MD-PhD or PhD	MD	MD-PhD or PhD	MD	MD-PhD or PhD
**Surgical faculty**	3,850	100%	90.1%	9.9%	87.6%	12.4%	85.2%	14.7%
Publications, median ± SD (Range)	3,850	100%	13 ± 29 (1–439)	23 ± 43 (2–288)	36 ± 40 (1–456)	75 ± 47 (6–239)	95 ± 127 (1–1938)	105 ± 113 (2–636)
Citations, median ± SD (Range)	3,850	100%	161 ± 516 (1–2105)	507 ± 1064 (19–6374)	543 ± 1706 (1–26158)	1553 ± 2008 (96–14101)	2200 ± 4446 (2–55118)	2516.5 ± 3842 (23–26315)
3-year citations, median ± SD (Range)	3,850	100%	57 ± 178 (1–2105)	191 ± 395 (15–2448)	175 ± 461 (1–6361)	175 ± 461 (1–6361)	473 ± 1215 (2–19986)	765 ± 1048 (23–6293)
H-index median ± SD (Range)	3,850	100%	6 ± 5 (1–36)	11 ± 7 (1–40)	11 ± 7 (1–61)	11 ± 7 (1–61)	21 ± 13 (1–89)	24 ± 14 (1–87)
**NIH funding**								
Current or Former funding,n, %	844	23.4%	16.8%	36.8%	20%	58%	34.3%	62.3%
**Divisions, n, %**								
Cardiothoracic Surgery	400	10.4%	83.5%	16.5%	86.2%	13.8%	86.5%	13.4%
General Surgery	1,875	48.7%	93.3%	6.8%	91.1%	8.9%	90.4%	9.6%
Acute Care Surgery	89	2.3%	95.8%	4.2%	97.5%	2.5%	86.7%	13.3%
General and Minimally Invasive	1,028	26.7%	91.5%	8.5%	91.6%	8.4%	90%	10%
Surgical Oncology	454	11.8%	93.5%	6.5%	93.5%	6.5%	87.5%	12.5%
Trauma/Critical Care	304	7.9%	97.5%	2.5%	95.7%	6.3%	94%	6%
Pediatric Surgery	370	9.6%	93%	7%	93%	7%	94.5%	5.5%
Plastic Surgery	374	9.7%	95.7%	4.3%	93.4%	6.6%	96.6%	3.4%
Science/Research	150	3.9%	17.2%	89.7%	25%	75%	12%	88%
Transplant	392	10.2%	83.5%	16.5%	87.9%	12.1%	79.4%	20.5%
Vascular surgery	289	7.5%	95%	5%	94%	6%	96.9%	3.1%

The presence of a Ph.D. degree had a positive effect on the individual academic performance for surgical faculty members. This positive effect of a Ph.D. was greater for faculty in the assistant and associate professor rank. Within these ranks, faculty with Ph.D.s had approximately two times as many publications and three times as many citations (**Table 2**). Among assistant professors, publications ± SD, citations ± SD were; 23 ± 43, 507 ± 1064 for PhD faculty compared with 13 ± 29, 161 ± 516 for MDs, p < 0.001. Among associate professors, these figures were; 75 ± 47, 1553 ± 2008 compared with 36 ± 40, 543 ± 1706, (p < 0.001). Full professors with Ph.D.s had a small but significantly higher number of publications and citations compared with their non-PhD rank equivalents. The median publications, citations for professors with PhDs was 105 ± 113, 2516 ± 3842 and those for MDs were 95 ± 127, 2200 ± 4446, p < 0.05. This increased academic productivity for those possessing a Ph.D. degree was also associated with a higher proportion of faculty with current or former NIH funding. Within each rank, faculty with PhDs were two times as likely to have had current or former NIH funding **(Table 2).** Among assistant professors, 36.8% of the Ph.D. faculty had current or a history of NIH funding compared with only 16.8% of the MDs. Similarly, higher percentages of NIH funding among PhD faculty were seen among associate professors (58% vs. 20%) and full professors (62.3% vs. 34.3%).

### Impact of NIH funding on research productivity among surgical faculty (Table 3)

Overall, 23.4% of faculty had current or former NIH funding, of which 9.4% had R01, P01, or U01 NIH grants (R01/P01/U01) and 13.8% were funded through other smaller funding mechanisms (including F32, K08, and R series awards). History of NIH funding correlated with significantly increased academic productivity. For faculty with R01/P01/U01 funding, the median publications ± SD, citations ± SD were; P: 109 ± 165, C: 3026 ± 5120 compared with P: 56 ± 107, C: 1257 ± 3763 for other smaller NIH grants, and P: 27 ± 66, C: 415 ± 2215 for faculty with no NIH funding.

**Table 3:** Academic output by type of current or former NIH funding and distribution of NIH funding by surgical divisions.

Parameter	Number (n) among all faculty	Percent% among all faculty	NIH R01, P01, U01 grants	Other NIH grants	No current or former NIH funding
Surgeons, n, %	3,859	100%	366, 9.5%	539, 14%	2,945, 76.5%
Scholarly output	3,850	100%			
Publications, median ± SD	3,850	100%	109 ± 165	56 ± 107	27 ± 66
Citations, median ± SD	3,850	100%	3026 ± 5120	1257 ± 3763	415 ± 2215
3-year citations, median ± SD	3,850	100%	744 ± 1035	340 ± 844	130 ± 846
H-index	3,850	100%	27 ± 15	16 ± 13	10 ± 10
Distribution of funding among divisions					
Cardiothoracic Surgery	400	10.4%	10.9%	19.1%	70%
Cardiac surgery	146	3.8%	9.5%	10.2%	80.3%
Thoracic surgery	254	6.6%	11.8%	24.4%	63.8%
General Surgery**	1,875	48.7%	7.1%	13.0%	79.9%
Acute Care Surgery	89	2.3%	8.4%	20.2%	71.4%
General and Minimally Invasive	1,028454	26.7%11.8%	7.6%	10.9%	81.5%
Surgical Oncology	304	7.9%	10.2%	18.2%	71.6%
Trauma/Critical Care	89	2.3%	4.3%	8.6%	87.1%
Pediatric Surgery	370	9.6%	11.3%	9.6%	79.1%
Plastic Surgery	374	9.7%	2.8%	10.6%	86.6%
Science/Research	150	3.9%	25.3%	31.3%	43.4%
Transplant	392	10.2%	19.3%	15.4%	65.3%
Vascular surgery	289	7.5%	7.2%	13.0%	79.8%

**Cardiothoracic surgery includes cardiac and thoracic surgery; General surgery includes acute care surgery, general and minimally invasive, surgical oncology, and trauma and critical care

Subset analysis **(Table 3)** revealed considerable variation in the proportion of faculty in each division that had some history of NIH funding. While 57% of faculty in the science and research division had current or past NIH funding, this number was considerably lower for faculty in other divisions. The next highly funded specialties for any history of NIH funding were transplantation (35%), cardiothoracic surgery (30%), and surgical oncology (28%). Specialties with the smallest proportion of NIH funded faculty included trauma/critical care and plastic surgery (13% each).

Analysis of individual sub-specialties demonstrated that faculty with current or past NIH funding consistently had higher numbers of publications and citations compared with their non-NIH funded counterparts **(Table 4)**. Among faculty with NIH funding, those with R01/P01/U01 funding also had significantly higher academic productivity as measured by total publications and citations compared with other smaller NIH grants. This correlation was observed for each specialty. Overall, faculty from cardiothoracic and vascular surgery with NIH R01/P01/U01 funding had the highest median numbers of publications (140 papers) and transplant surgical faculty had the highest numbers of citations (3775).

**Table 4:** Subset Analysis of scholarly output by type of current or former NIH funding. Cardiothoracic surgery includes cardiac and thoracic surgery; General surgery includes acute care surgery, general and minimally invasive surgery, surgical oncology, and trauma and critical care.

Parameter	n	(%)	NIH R01, P01, U01 grants		Other NIH grants		No current or former NIH funding	
	3,850	100%	Publicationsmedian ± SD	Citations median ± SD	Publicationsmedian ± SD	Citations median ± SD	Publications median ± SD	Citations median ± SD
Divisions								
**Cardiothoracic Surgery**	400	10.4%	137 ± 118	3518 ± 3194	60 ± 81	1220 ± 3006	35 ± 74	599 ± 2768
Cardiac surgery	146	3.8%	199 ± 121	4402 ± 2626	59 ± 75	1232 ± 1917	53 ± 87	662 ± 3803
Thoracic surgery	254	6.6%	124 ± 121	3238 ± 4039	60 ± 83	1220 ± 3227	31 ± 62	592 ± 1714
**General Surgery**	1,875	48.7%	99 ± 126	2797 ± 4950	60 ± 97	1536 ± 3969	25 ± 68	400 ± 2519
Acute Care Surgery	89	2.3%	99 ± 82	3252 ± 1898	22 ± 54	988 ± 1358	22 ± 39	283 ± 1223
General and Minimally Invasive	1,028454	26.7%11.8%	96 ± 149	2349 ± 6030	60 ± 96	1601 ± 2772	25 ± 66	423 ± 2364
Surgical Oncology	304	7.9%	104 ± 83	3342 ± 3461	81 ± 104	1830 ± 5402	32 ± 82	470 ± 3212
Trauma/Critical Care	89	2.3%	121 ± 92	2296 ± 3198	60 ± 216	1200 ± 5623	16 ± 58	236 ± 2018
Pediatric Surgery	370	9.6%	92 ± 99	2331 ± 3211	40 ± 84	535 ± 2456	30 ± 65	444 ± 1787
Plastic Surgery	374	9.7%	124 ± 108	3569 ± 3993	28 ± 199	62 ± 4883	22 ± 45	284 ± 1096
Science/Research	150	3.9%	78 ± 70	2288 ± 1814	53 ± 54	1769 ± 2634	46 ± 64	867 ± 1577
Transplant	392	10.2%	113 ± 272	3775 ± 7769	63 ± 139	1381 ± 4775	46 ± 90	794 ± 2546
Vascular surgery	289	7.5%	140 ± 87	3251 ± 1815	44 ± 96	767 ± 1702	32 ± 57	440 ± 1748

### Academic metrics by total departmental NIH funding amounts

Publications and citations were next compared to University-based departmental NIH rank. Overall, analysis of publications and citations in pooled groups of 10 by NIH-funding rank revealed an inflection point at rank 21–30. The median numbers of publications for institutions ranked 1–10 through 21–30 was 43 with minimal variation in numbers of citations (705–825). However the median ± SD publications/citations for rank 31–40 were 32 ±60/592 ± 2966 and these numbers for departments ranked 41–50 were 34 ± 99/616 ± 3274. Analysis of the academic output of different specialties in these of the institution also revealed considerable variation in individual publications and citations **(Table 5).**

**Table 5:** Subset analysis of scholarly output of surgical faculty by university NIH-funding rank groups. Cardiothoracic surgery includes cardiac and thoracic surgery; General surgery includes acute care surgery, general and minimally invasive surgery, surgical oncology, and trauma and critical care.

Parameter	n	(%)	Rank 1–10	Rank 11–20	Rank 21–30	Rank 31–40	Rank 41–50
			Publications, Citations (median ± SD)	Publications, Citations (median ± SD)	Publications, Citations (median ± SD)	Publications, Citations (median ± SD)	Publications, Citations (median ± SD)
Overall			43 ± 112	43 ± 97	43 ± 107	30 ± 61	26 ± 80
			825 ± 3509	709 ± 3202	(825 ± 4178)	(528 ± 2047)	(363 ± 2319)
Divisions							
Cardiothoracic Surgery	408	12.3%	50 ± 78	66 ± 107	74 ± 112	32 ± 60	34 ± 99
			1105 ± 2481	1063 ± 3313	1185 ± 3474	592 ± 2966	616 ± 3274
General Surgery	1538	48%	36 ± 85	43 ± 91	50 ± 106	30 ± 61	24 ± 84
			650 ± 3070	775 ± 3446	1027 ± 5010	486 ± 2130	375 ± 2549
Acute Care Surgery	76	2.3%	17 ± 40	132 ± 69	52 ± 88	30 ± 41	15 ± 33
			451 ± 1363	1348 ± 1944	1345 ± 2060	563 ± 1304	181 ± 999
General and Minimally Invasive	828	25.8%	40 ± 88	33 ± 84	50 ± 82	26 ± 61	25 ± 95
			807 ± 2767	589 ± 3784	1037 ± 3682	499 ± 1767	401 ± 2939
Surgical Oncology	371	11.5%	51 ± 93	42 ± 89	51 ± 123	43 ± 73	31 ± 41
			1179 ± 4300	690 ± 2439	999 ± 6194	776 ± 3043	481 ± 1420
Trauma/Critical Care	263	8.2%	16 ± 55	37 ± 75	48 ± 198	17 ± 43	14 ± 38
			164 ± 1779	464 ± 2031	1200 ± 6034	218 ± 1051	275 ± 1452
Pediatric Surgery	311	9.7%	34 ± 83	35 ± 48	41 ± 56	36 ± 44	23 ± 66
			574 ± 2708	555–1017	721 ± 1645	721 ± 1645	303 ± 1158
Plastic Surgery	317	9.9%	23 ± 89	41 ± 56	17 ± 199	24 ± 52.9	17 ± 32
			398 ± 1425	479 ± 1414	351 ± 5550	344 ± 1411	200 ± 706
Science/Research	96	3%	61 ± 58	61 ± 58	84 ± 68	53 ± 110	38 ± 42
			1628 ± 2374	1222 ± 1737	1982 ± 1026	1982 ± 1026	871 ± 1504
Transplant	303	9.4%	65 ± 219	77 ± 151	54 ± 76	40 ± 59	35 ± 88
			1873 ± 6371	2155 ± 3574	1069 ± 2246	906 ± 1750	598 ± 3191
Vascular surgery	222	6.9%	39 ± 64	32 ± 77	44 ± 67	31 ± 44	33 ± 123
			819 ± 2164	443 ± 1612	980 ± 3020	579 ± 1069	376 ± 2402

Overall, there was a decreasing numbers of total publications at lower ranking departments of surgery; however, there were some exceptions by specialties. The median publications ± SD and citations ± SD for acute care surgery division for institutions ranked between 11–20 was P: 132 ± 69, C: 1348 ± 1944, which was dramatically higher than the performance for this division among other rank subgroups. There was a linear decline in the numbers of publications and citations after the rank group 21–30 for all specialties. For example, within general surgery where the numbers of median publications decreased from 50± 106, 30 ± 61, 24 ± 84, and the corresponding citations decreased from 1027 ± 5010, 486 ± 2130, 375 ± 2549 in the rank groups 21–30, 31–40, and 41–50 respectively **(Table 5).**

### The 10 most-cited-faculty members within a department are responsible for majority of the academic output in surgical departments

**Fig 2:**
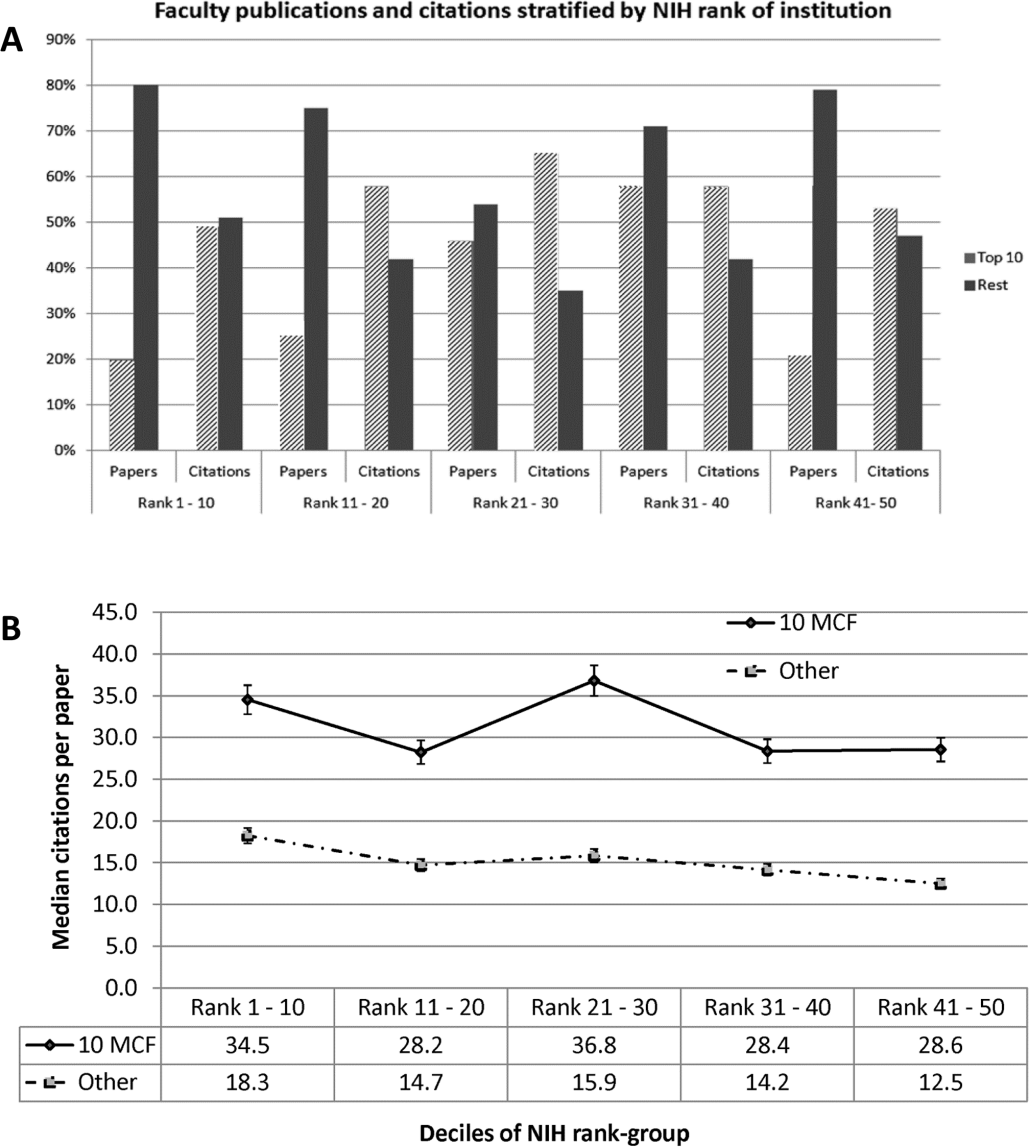
A Bar chart comparing between 10 most cited faculty and all other faculty, grouped by deciles of rank of the institution by NIH funding. Indicated for rank-group are the percentages of all publications, citations towards which the faculty contributed. The top-10 faculty contribute between 20% and 46% of publications and 49% to 65% of all citations in a department. B Comparisons between 10 most cited faculty and all other faculty regarding mean numbers of citations per paper, grouped by rank of the department of surgery by NIH funding.

Faculty members at each institution were ranked by their total numbers of citations. They were then divided into groups of 5, 10, and 20 most cited faculty (MCF). The median numbers of publications and citations were calculated for each group. Cut point analysis revealed that 10 individuals was the minimum number of faculty that were needed to achieve at least half of the citations in each NIH funded department of surgery rank group **(Fig 2A).** This number was tested over the entire dataset; there were small institutional variations and this number varied from 8 to 12 for most institutions. The 10 most cited faculty in the rank group 1–10 accounted for 20% of the publications and 49% of the citations. The group of 10 faculty (MCF) accounted for higher numbers of publications and citations at the lower ranking institutions. MCF accounted for 25% of the publications in rank 11–20, and 45% of the publications in rank 21–30 institutions. The proportion of citations that the 10 MCF also increased to 58% in rank 11–20, and 65% in rank 21–30 **(Fig 2A).** Three year citations similarly showed little difference relative to total citations (data not shown).

Not unexpectedly, the academic output for the 10-MCF was highest at the top 10 ranked departments **(Table 6).** Among the 10- MCF, there was a step-wise decrease in the publications and citations in the lower ranked departments of surgery. The median publications, citations for the 10-MCF professors at 10 best NIH funded departments of surgery were P: 249 ± 229, C: 7747 ± 6846 and these decreased to P: 129 ± 93, C: 3243 ± 3307 in the departments of surgery ranked 41–50.

**Table 6:** Scholarly output of top 10 faculty at each institution stratified by the institutional NIH funding rank.

			Publications ± SD, Citations ± SD				
Parameter			Rank 1–10	Rank 11–20	Rank 21–30	Rank 31–40	Rank 41–50
**Top 10 Faculty**			216 ± 226,	214 ± 154	187 ± 168	117 ± 80	120 ± 148
			7282 ± 6599	5977 ± 5697	6923 ± 6578	3629 ± 3510	3188 ± 4723
**Other faculty**			36 ± 53	36 ± 56	31 ± 53	23 ± 34	21 ± 38
			650 ± 1357	495 ± 1244	571 ± 1296	353 ± 728	282 ± 774
Academic Rank, Top 10 Faculty	Assistant professor	P	121 ± 312	288 ± 112	-	74 ± 74	75 ± 133
		C	5340 ± 6927	8168 ± 3881		2721 ± 2578	2786 ± 856
	Associate Professor	P	82 ± 60	108 ± 50	76 ± 17	53 ± 51	135 ± 137
		C	5494 ± 4152	3364 ± 1646	3156 ± 1243	2006 ± 7341	3037 ± 6010
	Professor	P	249 ± 229	229 ± 160	193 ± 168	136 ± 81	129 ± 93
		C	7747 ± 6846	6181 ± 6118	7746 ± 6639	3671 ± 2904	3243 ± 3307
Degrees, Top 10 faculty	MD	P	217 ± 229	179 ± 125	188 ± 175	114 ± 83	116 ± 107
		C	7447 ± 6713	4987 ± 5228	7139 ± 6760	3438 ± 3777	3188 ± 3659
	PhD.	P	158 ± 139	186 ± 161	148 ± 79	113 ± 37	132 ± 200
		C	5913 ± 3697	4793 ± 3462	3023 ± 4984	4845 ± 1464	3319 ± 3172
	MD., PhD.	P	410 ± 297	229± 90	178 ± 102	145 ± 89	161 ± 336
		C	15270 ± 8393	5870 ± 2086	9369 ± 4796	3460 ± 2428	3162 ± 12239
NIH funding, Top 10 faculty	NIH R01/U01/P01	P	259 ± 344	241 ± 207	187 ± 129	112 ± 73	213 ± 211
		C	8447 ± 9633	7351 ± 6655	5385 ± 2527	4478 ± 2068	5630 ± 6676
	Non–R01 funding	P	208 ± 147	198 ± 141	193 ± 247	142 ± 106	119 ± 149
		C	7769 ± 4962	6629 ± 4530	7116 ± 8443	3671 ± 4592	2653 ± 5275
	No NIH funding	P	183 ± 109	186 ± 127	171 ± 138	118 ± 75	112 ± 77
		C	5487 ± 3253	5336 ± 5410	7320 ± 6202	3059 ± 3569	2453 ± 2604

P = median publications

C = median citations, with standard deviations

Additionally, the 10 MCF with both M.D. and PhD degrees had the highest academic output. This effect was most pronounced at the 10 best ranked departments of surgery where the median publications, citations for M.D. Ph.D.s were 410 ± 297, 15270 ± 8393 compared with 217 ± 229, 7447 ± 6713 for MDs, and 158 ± 139, 5913 ± 3697 for PhDs **(Table 6).**

Analysis of the difference between the 10-MCF and all other faculty also revealed a considerable difference in the mean numbers of citations per paper, indicating higher impact publications. Publications for the 10-MCF were cited at least twice as many times as other faculty **(Fig 2B).** The mean citations per publication were 34.5 for the 10-MCF compared with 18.3 (p < 0.05) for other faculty at the top 10 NIH funded departments of surgery. Although, these figures decreased linearly at lower ranking departments of surgery, the gap in publication impact between the 10-MCF and other faculty did not go away. The mean citations per publication decreased to 28.6 for the 10-MCF and 12.5 (p < 0.01) for other faculty at the rank 41–50 departments of surgery **(Table 6).**

### The NIH funding among the top 10 MCF accounts for majority of funding for the department of surgery (Fig 3)

**Fig 3:**
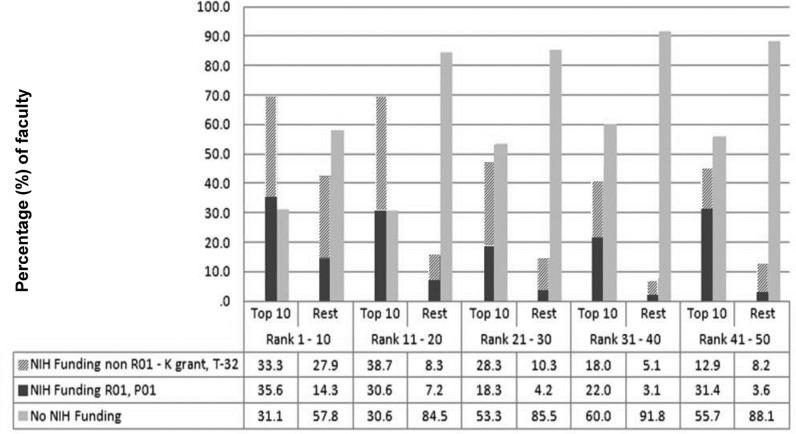
Extramural funding characteristics of the top cited faculty. Comparison of the (%) of faculty that are NIH funded between top-10 faculty and the other faculty in rank-groups by rank of NIH funding. The (%) that are NIH funded is indicated by Blue/Red bar, and (%) with no NIH funding are indicated by the green bar. This figure indicates that the top-10 faculty are more than two times likely to have any form of current/former NIH funding in every rank-group except the highest ranked institutions by NIH funding (Rank 1–11). In this group they are 50% more likely to have NIH funding than the “other faculty”.

There was considerable disparity regarding the proportion of faculty that were NIH funded when comparing 10-MCF and the other faculty. Most of the 10-MCF at the top 20 departments of surgery had current or former NIH funding. At departments of surgery ranked 1–10, 68.8% of the 10-MCF had current or former NIH funding compared with 42.2% of the other faculty (p < 0.001) and 69.3% of the 10-MCF faculty had a history of NIH funding in the rank 11–20 institutions compared with 15.5% of the other faculty (p < 0.001). In institutions ranked below 21–30, the 10-MCF were more than three times likely to have had NIH funding as compared with other faculty **(Fig 3).**

## Discussion

Research publications and impact of scholarly work are two of the most important measures of faculty accomplishment in academic medicine [8,9][28–30]. Such individual metrics are highlighted in numerous publically available university appointment and promotion guidelines [8,9][11][31] in both in the United States and Europe. Within departments of surgery, as in other clinically focused departments, in addition to undertaking research, faculty need to meet a variety of expectations including patient care, and teaching of multiple trainee groups in addition to undertaking research,. How to optimize each of these endeavors remains a clear challenge for both individual surgical faculty and leadership. Herein, we sought to better understand a current picture of academic productivity in American surgery as well as the apparent academic drivers within a department. To our knowledge, this manuscript is the first and most comprehensive overview of academic productivity at top university-based and hospital-based departments of surgery. In providing this detailed academic productivity overall, as well as within surgical sections and divisions, it provides metrics that can be useful for comparison and setting benchmarks for individuals, within respective sections, divisions and surgical departments.

Our data demonstrates that there is wide variation among different subspecialties with regard to their academic contribution to a department of surgery. Overall, divisions of science/research, transplantation, cardiothoracic surgery, and surgical oncology are the top four specialties with regard to high academic productivity, as measured by numbers of publications, citations, and NIH funding per faculty member. There is also great variability regarding academic productivity both between faculty members within a department and between departments of surgery. In this dataset, no significant difference concerning median publications or citations between departments of surgery that were ranked up to the top 30 NIH funded departments was observed. After 30, however, there was an aggregate drop in both the number of publications and citations.

We have also identified subsets of faculty expected to be high academic producers. Increased overall productivity is observed among those with advanced graduate degrees, higher academic rank, positions of administrative leadership, and within certain sections or divisions. Successful NIH funding at the faculty level is also correlated with the number of publications and citations, suggesting that successfully pursuing increased funding may likely also increase publications and citations, which in turn may associated with increased rates of subsequent successful NIH funding as well. This contradicts the conclusions of Jacob et al who have suggested that NIH grant awards only have a small effect on subsequent research productivity [2].

Another unique finding of this study is that the majority of academic productivity across sub-specialties in surgery lies in a relatively small number of faculty members. Our data demonstrate that approximately 10 faculty members, termed the MCF, contribute more than half of the citations and major grants within a department of surgery. Thus, for any department of surgery, its academic enterprise may be largely considered the work of a small fraction of the faculty, typically, 10 people of an average department size of 76 (13%). Identifying and supporting the MCF by encouraging them to lead in development of departmental research activities, and having them mentor newer faculty may allow optimal leveraging of research resources. Although there may be additional academically high producers, and the precise number 10 may vary somewhat from institution to institution, these individuals appear largely to determine the academic metrics for a particular department. A retrospective analysis of NIH funding for departments of surgery and medicine noted a significantly lower rate of increase in NIH funding for departments of surgery from 1992–1999 [32]. Other studies have also identified that NIH funding to surgical faculty is declining relative to non-surgical faculty [1][33]. In this era of increasing budgetary pressures and contracting extramural funding, the departmental support of the MCF will also likely better protect the core academic enterprise. Our findings also have implications for departments that seek to rise in NIH-funded departmental rankings. These data demonstrate that the academic performance of the 10 MCF group is highest in the top-10 NIH funded departments of surgery and gradually decreases with lower NIH funded ranks of the department, indicating that these faculty are able to better utilize resources towards successful academic performance. Furthermore, our data suggests that larger faculties may not be academically better, rather a smaller group in theory as few as 10 people, could define the best ranked department by academic metrics.

These data concerning individual faculty member’s publications, citations, and NIH funding were collected at the same time in order to interpret meaningfully, correlations that would potentially exist between scholarly metrics and NIH funding. The time-period of data collection for both the academic metrics and NIH funding were January 2014 to July 2014. It is anticipated that this relatively short duration of data collection resulted in minimal discrepancies between publications, citations, and NIH funding for faculty members.

There are a number of limitations regarding this study particularly in regards to available data sources. Numerous additional metrics are available and might have allowed for a clearer analysis. For example, authorship position was not considered in this analysis. Furthermore, although two sources for NIH funding were queried, due to the 6 months required to collect this data, some of the funding history may have changed. As well, errors in data collection, particularly around difficult to navigate departmental websites or common names may also have resulted in missed faculty or incorrect attributions. In order to minimize errors in data collection, we used stringent data management and two-person verification.

The authors acknowledge that academic output is not the only measure of academic success. Clinical productivity may be an important confounder in this analysis. Another important limitation of this study is that it is unable to account for clinical productivity of the faculty members in this dataset. Some anecdotal evidence however can be found among these faculty members, in that there are several examples of well recognized clinically productive faculty members with excellent academic publishing records. Furthermore, in the current climate of RVU based physician compensation, departmental expectations of clinical productivity are likely higher and protected time for research, considerably less. This in turn may help ameliorate the effect of clinical productivity on diminished research output.

These data also do not support any specific recommendations regarding junior unfunded faculty members in a department of surgery. A follow-up study after a 10–15 year period will help to better characterize the factors that promote a successful career trajectory for junior faculty, and the effect this has on the academic output of the departments of general and subspecialty surgery.

## Conclusions

This study provides a broad overview of the academic performance of general surgical subspecialties across the highest NIH funded departments of surgery. Cardiothoracic surgery, transplantation, and science/research divisions are the highest performers concerning publications and citations per faculty member. This study also identified important parameters, and the magnitude with which they predict successful NIH funding such as leadership positions, and PhDs or MD/PhDs. The presence of successful NIH funding is also associated with significantly higher research productivity. All NIH grants did not correlate with similar levels of academic success and the history of NIH R01/U01/P01 grants was associated with the greatest academic output. Finally, the identification of the MCF in the department is important, as the advancement of these faculty members drives the research performance of the entire department. With the current down trend in NIH funding for departments of surgery, the identification, promotion and retaining of the MCF may represent the best strategy towards overall departmental research success and NIH funding.

## Supporting Information

S1 File Supporting Information. (PLOS 1 Dataset deidentified Valsangkar Koniaris 2015.xlsx) This table includes the data used from institutional websites, SCOPUS, NIH Reporter and Grantome databases and lists the demographic details, academic metrics, and NIH funding information for the faculty members described in this study.
(XLSX)
